# Ex-Situ Evaluation of Commercial Polymer Membranes for Vanadium Redox Flow Batteries (VRFBs)

**DOI:** 10.3390/polym13060926

**Published:** 2021-03-17

**Authors:** Nana Zhao, Harry Riley, Chaojie Song, Zhengming Jiang, Keh-Chyun Tsay, Roberto Neagu, Zhiqing Shi

**Affiliations:** Energy, Mining & Environment Research Centre, National Research Council Canada, 4250 Wesbrook Mall, Vancouver, BC V6T 1W5, Canada; Harry.Riley@nrc-cnrc.gc.ca (H.R.); Chaojie.Song@nrc-cnrc.gc.ca (C.S.); Zhengming.Jiang@nrc-cnrc.gc.ca (Z.J.); Ken.Tsay@nrc-cnrc.gc.ca (K.-C.T.); Roberto.Neagu@nrc-cnrc.gc.ca (R.N.)

**Keywords:** membrane, vanadium redox flow batteries (VRFBs), ex-situ evaluation, vanadium ion crossover, chemical stability, proton conductivity

## Abstract

Polymer membranes play a vital role in vanadium redox flow batteries (VRFBs), acting as a separator between the two compartments, an electronic insulator for maintaining electrical neutrality of the cell, and an ionic conductor for allowing the transport of ionic charge carriers. It is a major influencer of VRFB performance, but also identified as one of the major factors limiting the large-scale implementation of VRFB technology in energy storage applications due to its cost and durability. In this work, five (5) high-priority characteristics of membranes related to VRFB performance were selected as major considerable factors for membrane screening before in-situ testing. Eight (8) state-of-the-art of commercially available ion exchange membranes (IEMs) were specifically selected, evaluated and compared by a set of ex-situ assessment approaches to determine the possibility of the membranes applied for VRFB. The results recommend perfluorosulfonic acid (PFSA) membranes and hydrocarbon anion exchange membranes (AEMs) as the candidates for further in-situ testing, while one hydrocarbon cation exchange membrane (CEM) is not recommended for VRFB application due to its relatively high VO^2+^ ion crossover and low mechanical stability during/after the chemical stability test. This work could provide VRFB researchers and industry a valuable reference for selecting the polymer membrane materials before VRFB in-situ testing.

## 1. Introduction

Large-scale energy storage systems with super long lifespans are a key solution to effectively incorporating renewable energy sources (e.g., solar and wind power) into power systems. This has become a major focus of attention for the world in the battles against climate change. Redox flow batteries (RFBs) have been recognized as a promising candidate for efficient, large-scale energy storage because their energy and power capacity can be scaled up independently [1,2]. In particular, the all-vanadium redox flow battery (VRFB), among other redox chemistries, has received significant attention in both academic and industrial communities, primarily due to its avoidance of cross-contamination between the two half-cell electrolytes ascribed to the employment of vanadium as a single active element in both half-cells [3]. However, the cost is still the main issue preventing broad market penetration of VRFBs. Membranes have been identified as one of the most expensive stack components, taking up 37% of the total cost in a 250 kW VRFB stack using Nafion™ as a membrane [4]. Lack of membranes that have a low cost, high stability and excellent ion selectivity is one of the major hurdles preventing VRFB technology from large-scale commercialization [5].

Substantial efforts have been made to reduce the cost and improve the chemical stability and ion selectivity of membranes over the last few decades [6,7,8,9,10,11,12,13,14,15,16,17]. At the current stage, the perfluorinated membranes are the most widely used in VRFBs due to their great chemical stability in the vanadium electrolyte and high proton conductivity. Nafion™ is a premiere commercial perfluorosulfonic acid (PFSA) membrane used in VRFBs, which is a cation exchange membrane (CEM) discovered in the late 1960s by Walther Grot of DuPont and currently is a trademark of Chemours [18]. A large number of research works have been published recently to compare the performance of these traditional Nafion membranes in VRFB application [19,20,21,22]. Reed et al. [20] installed Nafion^®^ N115, Nafion^®^ NR-212, and Nafion^®^ NR-211 into 3-cell 1 kW stacks to evaluate their VRFB performances. The thinnest membrane, NR-212, is advisable for use at high current densities to serve the needs for a VRFB such as reduced cost, ease of handling, and high performance. A series of Nafion membranes, N112, N1135, N115, and N117, were also evaluated and compared by Jiang et al. [21]. They reported that N115 was the most suitable for VRFB applications due to its favourable trade-off between vanadium ion crossover and membrane resistance. The VANADion membrane is a brand-new composite PFSA membrane developed by Union Chemical Industrial Co., Ltd., for VRFB application. Compared to N115, Zhou et al. claimed that the VANADion membrane is a promising membrane candidate for VRFB due to its better energy efficiency (EE), higher electrolyte utilization and similar capacity retention [23]. However, all the PFSA membranes exhibit vanadium crossover and high cost issues regardless of their thickness, ion-exchange capacity (IEC) and reinforcement. Therefore, considerable efforts have been devoted to investigating hydrocarbon-based materials as an alternative solution to PFSA-based membranes driven by cost-effective mass production in the marketplace [9,10,11,12,13,14,15,16,17]. It is thought that hydrocarbon-based anion exchange membranes (AEMs) could suppress vanadium permeability due to the Donnan exclusion effect [12]; however, their chemical and mechanical stability in VRFB operational conditions and lackluster ionic conductivity make their viability questionable [24,25]. Hydrocarbon-based membranes were primarily developed for fuel cell applications; as such, there are limited publications about the use of commercial AEMs in VRFBs. Skyllas-Kazacos’s research group has extensively studied the chemical stability of the commercially available AEMs including New Selemion (type 2), Selemion AMV, New Selemion Type 3H and a sample from Tokuyama [26,27]. They found that swelling behavior has a significant effect on the degree of membrane degradation due to the oxidation of polymers by V^5+^ ions. Choi et al. also evaluated three commercial AEMs—Selemion APS, AHA and AFN—as separators in VRFBs [28]. More recently, the possibility of commercial Fumasep AEMs for VRFB application has also been examined by several research groups [29,30,31]. Cao et al. [29] evaluated the permeation rate of the different vanadium ions across Fumasep^®^ FAP-450 with a comparison to N115, and Nguyen’s group [30] examined FAP-450 with respect to vanadium ion uptake under both static equilibria and after cell operating conditions. Besides ion exchange membranes (IEMs), non-ionic porous membranes have also been tried for VRFB such as polyvinylidene fluoride (PVDF), polytetrafluoroethylene (PTFE), polybenzimidazole (PBI) and polyvinylpyrrolidone (PVP)-based porous membranes due to their excellent mechanical stability, high chemical resistance and low cost [32,33,34,35,36].

Many detailed studies have been conducted using ex-situ methods of characterization of various commercial membranes in VRFBs over the last few decades. These research works have different evaluation approaches and are under a diverse set of test conditions that are not easily comparable after a certain point. There is a high demand from VRFB developers and researchers to harmonize these evaluation methods, and establish the effective and appropriate evaluation/diagnostic tools to determine the most essential properties of membranes linked to VRFB performance. In this work, five (5) key properties, namely, chemical stability, mechanical strength, proton conductivity/resistance, vanadium ion permeability/ion selectivity, and swelling property, were identified as a set of ex-situ membrane assessment criteria to screen the membranes for VRFB application. Among them, membrane chemical stability was first-time evaluated by membrane tensile strength changes before and after soaking V^5+^ solution for ten days. Eight (8) current state-of-the-art, commercially available membranes made by PFSA and hydrocarbon polymers were selected and evaluated by the methods of ex-situ characterizations mentioned. The results were used to access the qualification of these membranes in the VRFB applications. This work could provide the VRFB researchers and industry a reference for selecting the membrane materials before the VRFB in-situ testing.

## 2. Materials and Methods

### 2.1. Materials

Eight (8) of the current state-of-the-art commercially available polymer membranes for VRFB application were selected for ex-situ evaluation, including three (3) PFSA dense membranes, one (1) reinforced PFSA membrane and four (4) hydrocarbon membranes. All the membranes were used directly without further treatment. The investigated membranes, including their trade name, manufacture (supplier), types, thickness and IEC, are listed in Table 1. More detailed information is provided in Table S1. The benchmark PFSA membrane, Nafion 212, is used as a baseline in this report. 

### 2.2. Ex-Situ Characterizations 

#### 2.2.1. Chemical Stability Testing 

The accelerated chemical stability of the selected membranes was tested by immersing the membrane samples into the V^5+^ solution prepared from an operating VRFB cell. Specifically, the chemical stability tests were conducted by soaking 3 × 3 cm membranes in 1.6 M V^5+^ solution with a 4 M total sulfate at 40 °C for 10 days. Oxidation of the membranes was monitored by VO^2+^ concentration change in the soaking solution, as VO^2+^ ions are released when oxidation of a membrane occurs by V^5+^ solution. The concentration of VO^2+^ in the soaking solution was periodically measured by UV–Vis spectroscopy (Varian 50 Conc UV–Visible Spectrophotometer) during the chemical stability test. To establish the calibration curve, mixtures of 0.1 M VO^2+^ solution and 0.1 M V^5+^ solution were prepared at different ratios. The absorbance was determined for each mixture at a wavelength of 760 nm at which the maximum absorbance of VO^2+^ takes place and the plot of the absorbance of mixtures of VO^2+^ solution and V^5+^ solution against the concentration of VO^2+^ was shown in Figure S1. It is worthwhile to mention that all of the membranes were directly used without any pre-treatment for this chemical stability test. Some of the degradation might be related to the oxidation of trace solvents or the exchangeable ions. 

#### 2.2.2. Membrane Mechanical Strength Measurement

Tensile strength of the selected membranes was measured before and after soaking membranes into V^5+^ solution for 10 days. The membranes after chemical stability testing were soaked into 2 M H_2_SO_4_ for one day before tensile strength measurements. The membranes were die-cut to a barbell shape using a standard ASTM D638-4 cutter. The tensile tests were conducted on a universal test machine (2000 R System, Test Resources) with a load sensitivity of 0.1 N at room temperature (RT, 21 °C) and in atmosphere (approximately 40% RH was controlled by the building air condition system and measured when testing). The strain rate used was 0.015 s^−1^. Triplicate measurements were conducted for each membrane. Wet state membrane samples were used for AHA, AMV and CMV membranes, while dry state membrane samples were used for the rest of the membranes. 

The measured tensile strength of the membrane before the chemical stability test is treated as the initial membrane mechanical strength, while the tensile strength measured after the chemical stability test is used for evaluating membrane chemical stability based on the mechanical property changes.

#### 2.2.3. Vanadium Crossover Measurement 

Vanadium crossover represents the diffusion of vanadium ions across the membrane, leading to coulombic efficiency (CE) drop, capacity decay and OCV decline in VRFB charge/discharge cycling. In order to determine the diffusion coefficients of the VO^2+^ ions penetrating through the selected membranes, a two-half diffusion cell (PermeGear, Inc., Hellertown, PA, USA) was employed [25]. The tested membrane was sandwiched between the two half diffusion cells and a pair of rubber gaskets. The membrane’s exposed area to the two half cells was 1.76 cm^2^. A total of 5 mL of 1 M VOSO_4_ in 2 M H_2_SO_4_ was placed in the left half-cell and 5 mL of 1 M MgSO_4_ in 2 M H_2_SO_4_ in the right half-cell, and each side was magnetically stirred at 200 RPM to prevent the concentration polarization. The MgSO_4_ solution was used to equalize the ionic strengths of the two solutions and to minimize any osmotic pressure effects. A measurement of the concentration of VO^2+^ ions was determined by UV–Vis spectroscopy (at RT), whereby 2 mL of the right-side solution was taken at selected time intervals. The extracted sample solution was returned to the right half-cell immediately after UV–Vis measurement. Prior to the experiment, a standard curve of VO^2+^ solutions of different concentrations was prepared and is shown in Figure S2. The peak absorbance of VO^2+^ ion was established at 760 nm and acts as a reference.

The crossovers of VO^2+^ ions across the selected membranes were compared and their diffusion coefficients of vanadium ions were calculated using Sun’s approach [25,37] by Equation (1):

(1) $$V_{B}\cdot \frac{dC_{B}\left(t\right)}{dt}=A\cdot \frac{D}{L}\left(C_{A}\left(t\right)-C_{B}\left(t\right)\right)$$

where *V_B_* is the volume of solution on the deficiency side (mL); *C_A_* and *C_B_* are the concentration of VOSO_4_ on the enrichment side and deficiency side (mol∙mL^−1^), respectively; *A* is the effective area of the membrane (cm^2^); *D* is the diffusion coefficients of VO^2+^ (cm^2^∙s^−1^); *L* is the thickness of the membrane (cm); *t* is the testing time (s). 

The concentration of VO^2+^ ions on the deficiency side is lower than that at the enrichment side; therefore, the change of VO^2+^ ion concentration at the enrichment side is minor. Equation (1) was modified to Equation (2):

(2) $$C_{B}=A\cdot \frac{C_{A}\left(0\right)\cdot D}{V_{B}\cdot L}\cdot t$$

The plot of *C_B_* versus time is linear and has a slope (*k*) equal to $A\cdot \frac{C_{A}\left(0\right)\cdot D}{V_{B}\cdot L}$, so the diffusion coefficient (*D*) can be calculated as Equation (3):

(3) $$D=\;\frac{k\cdot V_{B}\cdot L}{A\cdot C_{A}\left(0\right)}$$

#### 2.2.4. Membrane Ionic Conductivity Measurements and Area Resistance

A two-probe conductivity cell with two Pt strips (distance: 1.6 cm) was mounted on a Teflon block as both AC current injectors and voltage measurement probes were employed for membrane conductivity measurement [38]. A piece of 2.4 × 1.0 cm membrane was immersed in deionized water for 24 h to be fully hydrated before measuring and then sandwiched between the two Teflon blocks. The impedance measurement was conducted immediately following filling water into the open windows of the Teflon block to avoid membrane dehydration. A Solatron 1260 (Solartron Analytical, Farnborough, UK) impedance/gain-phase analyzer was used to measure the resistance. The applied perturbation amplitude of the AC signal was 10 mV RMS (root mean square) over a frequency range 10 MHz to 100 Hz. All measurements were performed at an ambient temperature of 21 °C. Membrane in-plane ionic conductivity in water (σ) was calculated by Equation (4):

(4) $$\sigma =\;\frac{L}{A\cdot R}$$

where *σ* is the ionic conductivity of the membrane (S∙cm^−1^), *L* is the length of the membrane between the potential probes (cm), *R* is the resistance (Ω) obtained from the high-frequency intercept of the semicircle with the real impedance axis, and *A* is the membrane cross-sectional area (cm^2^). For each sample, three pieces of membrane were tested to calculate the average ionic conductivity and standard deviation.

The membrane area resistance was also measured in 2 M H_2_SO_4_ in an H-cell, which was separated by a membrane with an effective area of 1 cm^2^. The cell was filled with 2 M H_2_SO_4_ in each half cell. Two carbon rod electrodes were connected with Solatron 1260 (Solartron Analytical) impedance/gain-phase analyzer to measure the area resistance over a frequency range from 100 to 1 MHz. The membrane was pretreated in a 2 M H_2_SO_4_ solution for 24 h. The membrane area resistance (R) is calculated by Equation (5):

R = A × (R_1_ − R_2_) (5)

where A is the membrane active area (cm^2^), R_1_ is the resistance of the cell with the membrane (Ω) and R_2_ is the resistance of the cell without the membrane (Ω).

The ion selectivity represents the ratio of ionic conductivity over vanadium ion permeability and was calculated by Equation (6).

(6) $$S=\frac{\sigma }{P}$$

where *S* is the ion selectivity of the membrane (S∙s∙cm^−3^); *σ* is the ionic conductivity of the membrane (S∙cm^−1^); *P* is the diffusion coefficients of VO^2+^ (m^2^∙s^−1^).

#### 2.2.5. Membrane Swelling Property Testing

The swelling of a membrane in vanadium electrolyte could affect VRFB performance and durability. Membrane swelling was measured through mass change and dimensional change before and after exposing the membrane to vanadium electrolyte solution. The weight and dimension of a piece of membrane (2 × 5 cm) was measured, then it was soaked into 18 mL of 1.6 M VOSO_4_ solution (with 2 M H_2_SO_4_) at RT for 24 h. The membrane was then removed from the solution and dried by Kimwipe. The weight and dimensions were re-measured. All the measurements were conducted at 21 °C with an RH of 40%.

The quantity of water and electrolyte uptake inside the membrane was determined by mass change according to Equation (7):

(7) $$Mass\;change\;\left(\%\right)=\frac{m_{wet}-m_{dry}}{m_{dry}}\cdot 100\%$$

where *m_wet_* and *m_dry_* are the mass of the membrane after and before soaking in VOSO_4_ solution, respectively.

The dimensional change of the membrane was calculated by Equation (8):

(8) $$Dimensional\;change\;\left(\%\right)=\frac{V_{wet}-V_{dry}}{V_{dry}}\cdot 100\%$$

where *V_wet_* and *V_dry_* are the volumes of the membrane after and before soaking in VOSO_4_ solution, respectively.

## 3. Results and Discussion

### 3.1. Membrane Chemical Stability 

**Figure 1:**
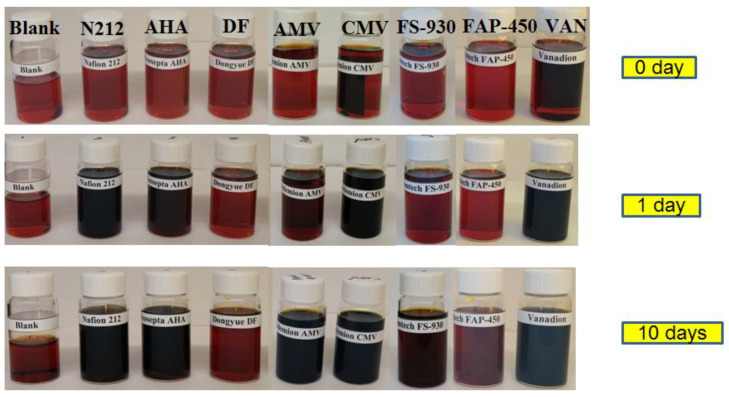
Electrolyte (1.6 M V^5+^ solutions in 4.0 M total sulfate) solutions and membrane samples after immersion with time.

An accelerated chemical stability test was carried out to screen the selected membranes by soaking the membranes in a solution containing 1.6 M V^5+^ and a total of 4.0 M SO_4_^2−^ at 40 °C. During the 10 days of immersion, the soaking solutions were monitored by both visual observation of the colour change in V^5+^ solution and UV–Vis spectroscopy. Figure 1 shows the photograph of the V^5+^ soaking solutions with membranes as well as blank solution (1.6 M pure V^5+^ fully understood solution without membrane) at 0 day, 1 day and 10 days. After membrane immersion for 1 day, the solutions with N212, AHA, AMV, CMV and VAN membranes began to turn dark, indicating the release of VO^2+^ ions in the soaking solutions, while the solutions with DF, FS-930 and FAP-450 membranes retained their original colour. Generally, the colour of pure the VO^2+^ ion and pure V^5+^ ion electrolyte is yellow and blue, respectively. When the membrane is oxidized by V^5+^ ions in the soaking solution, some V^5+^ ions with a yellow colour are reduced to VO^2+^ ions with a blue colour [27,39]. The observed colour changes in some V^5+^ solutions are possibly due to the generated blue colour from VO^2+^ ions, suggesting that N212, AHA, AMV, CMV and VAN membranes started degradation in the first day. However, there was no obvious colour change in the solutions containing DF, FS-930 and FAP-450 membranes, indicating that these three membranes were more stable than the other selected membranes in the first 24 h. After 10 days, all the solutions went to further darken (mixture of yellow and blue) except DF and FAP-450 membranes, implying that more V^5+^ ions were reduced upon reaction with the investigated membranes. In contrast, the colour of the soaking solutions of the vials containing DF and FAP-450 did not change with time, which was similar to that observed in the blank solution. This indicates that VO^2+^ ions in the immersion solution with DF and FAP-450 membranes were not visible and the membrane degradation rate was lower than others or no oxidation reaction occurred between membranes and V^5+^ electrolyte in these 10 days.

**Figure 2:**
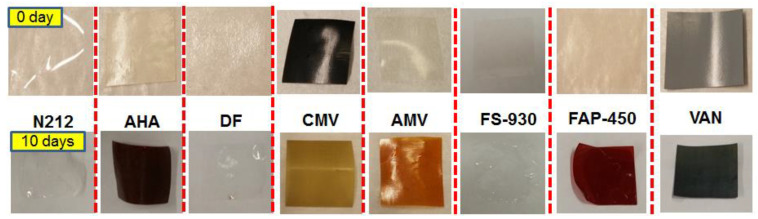
Photograph of membranes before (**top**) and after (**bottom**) chemical stability tests.

Figure 2 shows a photograph of each membrane before and after the chemical stability test, proving that all of the investigated membranes survived without any cracking after 10 days of soaking in V^5+^ solution at 40 °C. A colour change from light to dark for the AMV, AHA, FAP-450 and VAN membranes was noticed due to their absorbance of the electrolyte, while the colour changed from black to yellow for CMV, possibly ascribable to the oxidation reaction of V^5+^ ions with CMV membrane. Contrastingly, N212, DF and FS-930 kept their original respective colours. 

**Figure 3:**
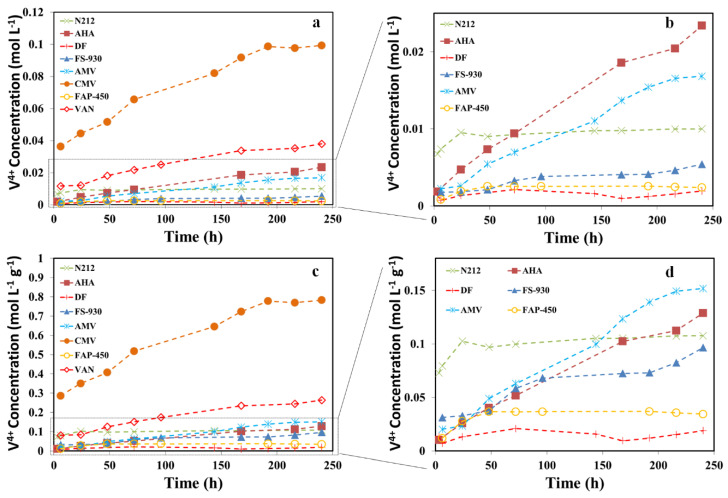
Membrane chemical stability carried by soaking the membranes in a solution containing 1.6 M V^5+^ and a total of 4.0 M SO_4_^2−^ at 40 °C for 10 days. (**a**) Concentration of V^4+^ ions in the electrolyte solutions with time for all the investigated membranes; (**b**) Zoomed-in curves of (**a**) without CMV and VAN; (**c**) Concentration of V^4+^ ions in the electrolyte solutions normalized by the mass of the membrane against time; (**d**) Zoomed-in curves of (**c**) without CMV and VAN.

Membrane degradation in VRFB is believed to be caused by the oxidation reaction with V^5+^, although the mechanism of this decay is still not fully understood [9,25,26,27]. Therefore, beside the visual observation of colour changes in soaking solutions as well as the tested membranes, the variation of VO^2+^ ion concentration in the soaking solutions was monitored by UV–Vis spectrophotometry, which in turn provided additional indication of the membrane degradation rate. Figure 3a presents the concentration of VO^2+^ ions in the soaking solutions against immersion time. Excluding CMV and VAN, Figure 3b is zoomed-in on a portion of Figure 3a, which could help to identify the differences in degradation rate for each membrane. In general, the amount of VO^2+^ ions increased with time for all the membranes, excluding DF and FAP-450 membranes, indicating that the membranes were likely oxidized as a result of the reduction of V^5+^ to VO^2+^ ions. As a comparison, the VO^2+^ ion concentration in the solution containing the CMV membrane was two orders of magnitude greater than that with baseline membrane (N212) on the 10th day of soaking. It revealed that the CMV membrane generated the largest amount of VO^2+^ ions and showed the worst stability among the selected membranes. DF and FAP-450 membranes display the best stability followed by FS-930 and then N212 (baseline). It is worth mentioning that the change in VO^2+^ concentration detected by UV–Vis for DF and FAP-450 membranes was insignificant, suggesting that there was very minor degradation for these two membranes in 10 days and being reflected by the lack of any colour change of the membrane in soaking solution, as shown in Figure 2. Moreover, VAN, AHA and AMV membranes were less stable than the baseline membrane. The chemical stability for the membranes is in the order of DF ≈ FAP-450 > FS-930 > N212 > AMV > AHA > VAN > CMV. Since the thickness (and density/weight) of the membranes is different, the concentration of VO^2+^ ions was normalized by the mass of the membrane for further investigation, shown in Figure 3c,d. Although the N212 baseline membrane shows the great degradation rate (only less than CMV in the first day), it levelled off and remained stable in the rest of the 9 days. The chemical stability of the membranes normalized by their weight is in the order of DF > FAP-450 > FS-930 > N212 > AHA > AMV > VAN > CMV. This is consistent with the observation of colour changes for the membranes and V^5+^ soaking solution after 10 days of chemical stability testing. Therefore, we believe that the normalized curves by the mass of the membrane would better reflect the chemical stability of the membranes. 

### 3.2. Membrane Mechanical Property 

Sufficient mechanical strength is one of the essential requirements for VRFB membranes, because these membranes suffer different stresses, from battery assembly to VRFB operation, including the high cell assembly pressure, various mechanical shocks, and dimensional changes from dehydration (dry) to hydration (wet) conditions. Most notably, membranes are required to maintain their mechanical strength in a long-term VRFB application to tolerate the harsh chemical environments (high concentration of supporting electrolyte (H_2_SO_4_) and highly oxidative reactivity of the electrolyte species). 

The intrinsic tensile strengths of the investigated membranes were measured before the chemical stability test at room temperature (RT, 21 °C) and 40% relative humidity (RH) and are summarized in Table 2. The PFSA membranes (N212, DF, FS-930), AMV and CMV displayed similar peak stress (~30 MPa), indicating that the intrinsic mechanical properties of these membranes are close. In contrast, VAN as a PFSA-based composite membrane exhibited a lower tensile strength than other PFSA membranes due to its unique dual-layered structure [23]. A thin, dense Nafion layer (∼20 μm) coated on a thick microporous layer (∼210 μm) allowed VAN to withstand a superior peak load, but relatively low peak stress. In addition, two hydrocarbon membranes, AHA and FAP-450 showed slightly higher peak stress (~40–50 MPa). Before the chemical stability test, the order of strength of the membranes is as follows: AHA ≥ FAP-450 > N212 ≈ DF≈ FS-930 ≈ CMV ≈ AMV > VAN.

After 10 days’ chemical stability test, all the tested membranes were visibly in good shape, which makes it difficult to identify the degradation done to the membranes. Therefore, the mechanical strength tests for the investigated membranes were conducted after the accelerated chemical stability test to evaluate the chemical/mechanical stabilities of the membranes. The ultimate peak stress losses of the selected membranes after chemical stability testing are presented in Table 2. It revealed that the FAP-450 membrane showed the highest tensile strength loss (40%) after chemical stability testing, although the changes of VO^2+^ concentration in the soaking solution for the FAP-450 membrane were imperceptible. The AHA membrane displays the lowest mechanical property loss (4%) followed by VAN and AMV. All the PFSA dense membranes had ~20% tensile strength losses after soaking in V^5+^ solution for 10 days, which presented a lower mechanical stability than that of hydrocarbon membranes. The thickness of the membrane samples is a factor possibly affecting the mechanical stability of the membranes due to the extended time that the V^5+^ ions attack the backbone of the inner polymer in thicker membranes. As one of the indicators for membrane chemical stability, the mechanical property loss of the investigated membranes after soaking in V^5+^ solution for 10 days is in the order of FAP-450 > CMV > DF > N212 > FS-930 > AMV > VAN > AHA. 

### 3.3. Membrane Vanadium Crossover 

**Figure 4:**
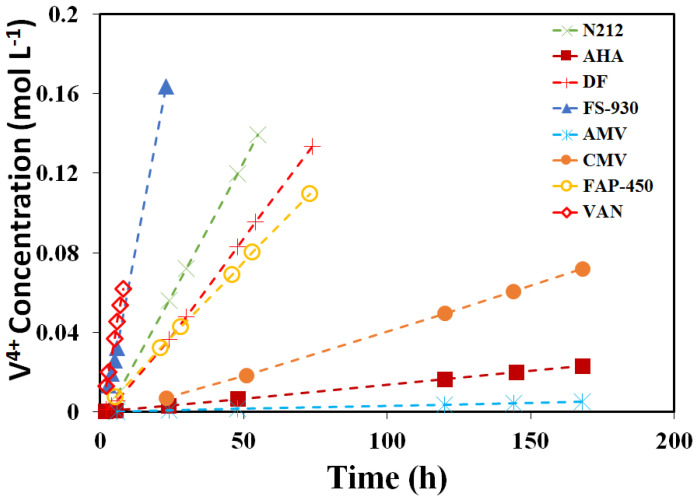
Concentration of the V^4+^ of eight (8) membranes at different times on the deficiency side.

It is inevitable that vanadium ions will pass through any of the membranes, leading to the reduction of CE and EE of a VRFB due to the overlapped hydrated ionic clusters and the complex interactions among different ions being transported in the membrane. In order to achieve durable and high-performance VRFB, a membrane possessing low vanadium ion crossover rate is desperately required. The crossovers of VO^2+^ ions across the selected membranes were compared and their diffusion coefficients of vanadium ions were calculated using Equation (3): 

Figure 4 shows the concentration of the VO^2+^ of each membrane against the time on the deficiency side. The calculated slopes (k) and diffusion coefficients (*D*) are listed in Table 3. In principle, the vanadium permeability across a membrane depends on the membrane properties of IEC, water uptake, thickness and microstructure [8]. Compared to PFSA membranes (9.2 × 10^−9^ to 1.62 × 10^−7^ cm^2^∙s^−1^ in Table 3), the AEMs showed lower diffusion coefficients (1.5 × 10^−8^ to 4.2 × 10^−7^ cm^2^∙s^−1^), due to the Donnan exclusion effect between the immobilized positively charged functional groups and the electroactive species. Meanwhile, the hydrocarbon membranes (3.5 × 10^−9^ cm^2^∙s^−1^ for CMV) more effectively suppress vanadium permeability than PFSA membranes for the sake of their microstructural distinction. It was reported that the fraction of ‘open’ ionic domains at the surface of the sulfonated hydrocarbon membranes, as measured by conductive probe microscopy, is much smaller than the surface of the PFSA membrane [40]. The diffusion coefficient of VO^2+^ ions is in the order of AMV < AHA < CMV < FAP-450 < DF < N212 < FS-930 < VAN.

### 3.4. Membrane Conductivity, Area Resistance and Ion Selectivity 

Membrane conductivity is another important factor determining the voltage efficiency (VE) of a VRFB since it controls the ohmic resistance of the cell. Table 4 shows the in-plane membrane ionic conductivity measured in water at RT. Generally, when the perturbation amplitude of the AC signal is applied by EIS, either protons or OH^−^ ions are transported within the ionic channel of a hydrated CEM or AEM. They can also migrate along the network formed by the hydrogen bond between water molecules and ionic functional groups in the membranes [41]. 

The degree of transport of ions through the membrane is defined as ionic conductivity. Specifically, the in-plane ionic conductivity measured in this work for CEM or AEM refers to proton conductivity or OH^−^ conductivity, respectively. The proton conductivities among PFSA dense membranes (NRE-212, FS-930 and DF) are comparable (60–84 mS∙cm^−1^) due to their analogous chemical structures, while the slight variances are mainly attributed to the difference in IEC (0.9–1.1 meq∙g^−1^). For CEMs, the PFSA-based dense membranes exhibited higher proton conductivities than the reinforced CEM (VAN, 0.54 mS∙cm^−1^) and the hydrocarbon-based CEM (CMV, 4.6 mS∙cm^−1^) on account of the differences in the physical and chemical structure of the membranes. Moreover, the proton conductivities of PFSA dense membranes are higher than the OH^−^ conductivities of AEMs because protons have higher mobility than hydroxyl ions (the intrinsic diffusion rate: H^+^ (9.31 × 10^−5^ cm^2^∙s^−1^) vs. OH^−^ (5.27 × 10^−5^ cm^2^∙s^−1^)) [7]. The OH^−^ conductivities for AEMs were in the range of 2.4–38 mS∙cm^−1^. In summary, the ionic conductivities of the membranes (σ) are in the following order: FS-930 ≈ N212 ≈ DF > FAP-450 > CMV > AMV > AHA > VAN.

The membrane area resistance was also measured in 2 M H_2_SO_4_ in an H-cell with two carbon rods as electrodes. PFSA-based membranes (N212, DF, FS-930) exhibited comparable area resistance with hydrocarbon membranes (FAP-450 and CMV), showing superior ion conductivity compared to VAN, AMV and AHA. It is worth mentioning that the thickness of the membrane is not taken into account in the calculation of membrane area resistance. In summary, the area resistance of each membrane is in the following order: DF ≈ N212 < FS930≈FAP-450 < CMV < VAN < AMV< AHA.

To minimize energy loss, it is essential for the membranes to acquire high ionic conductivity while preventing undesired ion crossover to ensure high VE and CE. Ion selectivity (S) is used to evaluate this sentiment and defined as the ratio of ionic conductivity (H^+^ or OH^−^) over vanadium ion permeability. Table 4 summarizes the calculated ion selectivity of eight (8) investigated membranes based on Equation (6). It is noteworthy that the conductivity used in Equation (6) was converted from the membrane resistance values measured in sulfuric acid using an H-cell, since the ion transportation direction for membrane resistance measurement is in the same direction as ion transporting in VRFB operation. AMV shows the highest ion selectivity (2.2 × 10^7^ S∙s∙cm^−3^) due to its extremely low vanadium crossover and acceptable conductivity. Although the PFSA-based membranes exhibited higher vanadium permeability, they showed great ion selectivity (0.3 × 10^6^–1.4 × 10^6^ S∙s∙cm^−3^) due to their relatively high proton conductivities. FAP-450 also displayed comparable ion selectivity (1.6 × 10^6^ S∙s∙cm^−3^) with PFSA-based membranes. In summary, the ion selectivity of each membrane is in the following order of magnitude: AMV > CMV≈AHA > FAP-450 ≈ N212 ≈ DF ≥ FS-930 > VAN. 

### 3.5. Membrane Swelling Property

It is crucial to minimize membrane swelling in the vanadium electrolyte in order to achieve good mechanical stability and provide a long VRFB lifetime, even though water uptake is required for ionic transporting through the membrane. On the other side, a high swelling ratio could result in high vanadium ion crossover. Table 5 and Table S2 show the dimensional changes and mass changes of the investigated membranes in V (IV) electrolyte solution (1.6 M VOSO_4_ solution in 2 M H_2_SO_4_) tested at 21 °C and RH 40%. In general, the dimensional and mass changes in VOSO_4_ solution for all the membranes are negligible (less than 10%) excluding FAP-450 and VAN membranes. The dimensional changes of VAN are minor; however, the mass change is very high (101%). It is understandable that the mass change is high due to the absorption of the electrolyte in the micro pores since VAN consists of a microporous layer and a dense Nafion layer. However, it is noticed that both the dimensional change and mass change for FAP-450 are high, specifically, ~40% and 60%, respectively. For a membrane material in VRFB applications, dimensional change is a more important property that should be taken into consideration, rather than mass change, because severe three-dimensional swelling could result in not only a decrease in mechanical strength, but also an escalated stress for the membrane during VRFB operation. For the dimensional change of the membranes investigated in this report, the order is CMV ≈ AMV ≈ FS-930 ≈ VAN ≤ DF≤ AHA≤ N212 < FAP-450. 

## 4. Conclusions

A membrane in a VRFB battery cell acts as a separator between the anode and cathode compartment to separate the active species, an electronic insulator, and an ionic conductor facilitating the transport of ions such as protons, or sulfate ions to maintain charge balance within the cell. For VRFB applications, the ideal membrane should have high ionic conductivity, low vanadium ion permeability, superior ion selectivity, good chemical stability, high mechanical stability and optimal water transportation properties as well as low cost. In this report, five membrane properties, including chemical stability, mechanical property, ion conductivity/area resistance and vanadium ion crossover (ion selectivity), as well as membrane swelling property, were identified as critical factors to determine the selection of membranes for VRFB.

Chemical stability for the selected membranes was evaluated in fully charged vanadium electrolyte (V^5+^ solution) and the membrane degradation rate was detected by the amount of VO^2+^ ions generated in V^5+^ solution and the changes in tensile strength before and after the chemical stability test. Of the membranes tested in this work, based on VO^2+^ concentration generated in V^5+^ solution, the CMV showed the lowest chemical stability, while FAP-450 and PFSA based membranes (DF, FS-930 and N212) showed excellent stability. However, according to the post-analysis of the tensile strength results, FAP-450 exhibited inferior stability. AHA and FAP-450 presented superior intrinsic tensile strength. Amongst the identified five membrane properties for membrane screening, ion selectivity is the most important factor on VRFB performance. The outstanding ion selectivity implies the best balance between ion conductivity and vanadium ion permeability, possibly leading to the greatest VRFB performance. AMV, CMV, AHA, FAP-450, and DF showed higher ion selectivity. For the dimensional changes, all of the membranes are comparable, except FAP-450.

To compare these eight (8) commercial membranes for VRFB application, each membrane candidate was ranked according to their ex-situ characterization results and is shown in Table 6. The best membrane for a certain property gets a rank of #1, counting one (1) point, and the worst gets a rank of #8, counting eight (8) points, which means that a higher point indicates that a membrane is less suitable for a VRFB. The overall property score for each membrane is the sum of the points of each property as an overall performance indicator to screen the membranes for VRFB applications. Due to the negligible difference in dimensional changes among the membranes (less than 10%), the point for all membranes in this property is treated the same as one (1) point, except FAP-450. Judging from the overall property score in Table 6, the fluorinated PFSA membranes (Nafion N212, Fumapem FS-930, and Dongyue DF) and the hydrocarbon AEMs (Selemion AMV, Neosepta AHA, and Fumapem FAP-450) are recommended as membrane candidates for further in-situ VRFB evaluation. On the other hand, VANADion™-20 VAN and Selemion CMV membranes are not recommended for VRFB applications based on the ex-situ characterization results in this report. The assessment and ranking in this work could provide a valuable reference for researchers and industry when selecting membrane materials for VRFB applications.

## Figures and Tables

**Table 1:** Investigated membranes for vanadium redox flow batteries (VRFBs).

Membranes			Company	Type	Thickness (µm)	IEC (mmol g^−1^)
1	N212	Nafion N212	Chemours (DuPont), Wilmington, DE, USA	CEM PFSA	50	0.9
2	FS-930	Fumapem FS-930	Fumatech GmbH, St. Ingbert, Germany	CEM PFSA	30	1.14
3	DF	Dongyue DF	Dongyue Chemical Ltd., Zibo, China	CEM PFSA	50	1.57
4	VAN	VANADion™-20	National Store (Ion Power), Umm Ramool, Dubai	CEM Reinforced PFSA	254	--
5	FAP-450	Fumasep FAP-450	Fumatech GmbH, St. Ingbert, Germany	AEM Hydrocarbon	50	2.18
6	AHA	Neosepta AHA	ASTOM Co. (Tokuyama), Yamaguchi, Japan	AEM Hydrocarbon	220	0.35
7	AMV	Selemion AMV	Asahi Glass Co., Ltd., Tokyo, Japan	AEM Hydrocarbon	110	1.6
8	CMV	Selemion CMV	Asahi Glass Co., Ltd., Japan	CEM Hydrocarbon	110	2.08

**Table 2:** Tensile strength of the selected membranes before and after chemical stability testing.

Sample Name		Peak Load (N)	Peak Stress (MPa)	Peak Stress Loss
N212	Before	4.3 ± 0.1	28.9 ± 0.5	20%
	After	3.5 ± 0.3	23.2 ± 1.9	
DF	Before	5.3 ± 0.2	29.3 ± 1.3	25%
	After	4.3 ± 0.3	21.9 ± 1.5	
FS-930	Before	2.7 ± 0.2	30.0 ± 2.0	18%
	After	2.2 ± 0.3	24.6 ± 3.1	
FAP-450	Before	5.5 ± 0.0	36.7 ± 0.2	40%
	After	3.7 ± 0.6	22.0 ± 3.6	
VAN	Before	12.5 ± 0.5	16.9 ± 0.7	6%
	After	11.7 ± 0.9	15.9 ± 1.2	
AHA	Before	34.3 ± 1.7	46.7 ± 2.3	4%
	After	28.8 ± 2.9	44.6 ± 4.6	
CMV	Before	9.9 ± 1.3	28.6 ± 3.8	32%
	After	6.7 ± 1.0	19.4 ± 2.9	
AMV	Before	9.3 ± 0.1	27.1 ± 0.2	7%
	After	9.0 ± 1.3	25.1 ± 3.5	

**Table 3:** Diffusion coefficients of VO^2+^ ions for different membranes.

Membranes	Slope/k (mol∙L^−1^∙h^−1^∙10^−4^)	Diffusion Coefficient (cm^2^∙s^−1^∙10^−7^)
AMV	0.3	0.0025
AHA	1	0.032
CMV	4	0.035
FAP-450	15	0.070
DF	18	0.092
N212	27	0.11
FS-930	76	0.18
VAN	82	1.62

**Table 4:** Ionic conductivity obtained in deionized (DI) water at RT and membrane area resistance measured in 2 M H_2_SO_4_ as well as the calculated ion selectivity data for the selected membranes.

Membranes	Ionic Conductivity σ (mS∙cm^−1^)	Area Resistance (Ω∙cm^2^)	Ion Selectivity (S∙s∙cm^−3^∙10^6^)
N212	70 ± 6	0.47	1.0
FS-930	84 ± 8	0.54	0.3
DF	60 ± 1	0.48	1.4
AMV	3.9 ± 0.0	2.08	21.8
CMV	4.6 ± 0.4	0.62	5.5
AHA	2.4 ± 0.2	2.39	2.8
FAP-450	38 ± 3	0.59	1.6
VAN	0.54 ± 0.01	1.23	0.1

**Table 5:** Dimensional changes and mass changes of membranes measured at 21 °C and RH 40% in 1.6 M VOSO_4_ solution (with 2 M H_2_SO_4_).

Membranes	Dimensional Changes (Volume Changes) (%)	Mass Changes (%)
N212	8 ± 4	6 ± 0.2
FS-930	3 ± 1	3 ± 1
DF	6 ± 3	4 ± 2
AMV	2 ± 2	9 ± 1
CMV	0 ± 1	1 ± 1
AHA	7 ± 1	8 ± 2
FAP-450	42 ± 8	57 ± 3
VAN	2 ± 1	101 ± 6

**Table 6:** Membrane ranking based on ex-situ property evaluation score.

Membrane		Membrane Property					Overall Property Score
	Chemical Stability (Rank)		Mechanical Property (Rank)	Ion Selectivity (Rank)	Area Resistance (Rank)	Dimensional Change (Rank)	
	[VO^2+^] Generated in V^5+^ Solution	Tensile Strength Loss					
N212	4	5	3	4	1	1	18
FS-930	3	4	3	4	3	1	18
DF	1	6	3	4	1	1	16
VAN	7	2	8	8	6	1	32
AMV	5	3	3	1	7	1	20
CMV	8	7	3	2	3	1	24
AHA	6	1	1	3	7	1	19
FAP-450	1	8	2	4	3	2	20

## Supplementary Materials

The following are available online at https://www.mdpi.com/2073-4360/13/6/926/s1, Figure S1: UV absorbance of V (IV)/V (V) solutions at different ratios. Figure S2: UV absorbance of VOSO_4_ in 2 M H_2_SO_4_ solutions at different concentrations. Table S1: IEC of the investigated membranes for VRFBs. Table S2: Length, width and thickness changes of membranes measured at 21 °C and RH 40% in 1.6 M VOSO_4_ solution (with 2 M H_2_SO_4_) comparing the dry membranes.
