# Supplementary material for: Ex-Situ Evaluation of Commercial Polymer Membranes for Vanadium Redox Flow Batteries (VRFBs)
Source: Polymers (Basel). 2021 Mar 17;13(6):926. doi: 10.3390/polym13060926 (PMC8002826; doi:10.3390/polym13060926)
Supplement: Supplementary file 1 [file polymers-13-00926-s001.pdf]

Supplementary Materials

Ex-situ **Evaluation of Commercial Polymer Membranes for Vanadium Redox Flow Batteries (VRFBs)**

Nana Zhao [1*], Harry Riley [1], Chaojie Song [1], Zhengming Jiang [1], Keh-Chyun Tsay [1], Roberto Neagu [1] and Zhiqing Shi [1*]

[1]Energy, Mining & Environment Research Centre, National Research Council Canada, 4250 Wesbrook Mall, Vancouver, B.C. V6T 1W5;

*Correspondence: Nana.Zhao@nrc-cnrc.gc.ca; Zhiqing.Shi@nrc-cnrc.gc.ca

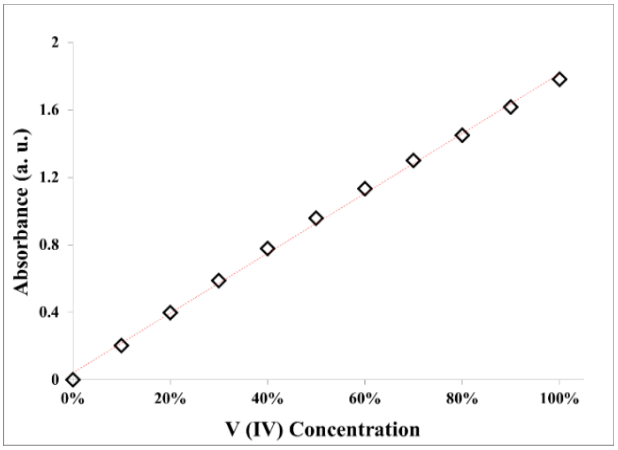

Figure S1. UV absorbance of V (IV)/V (V) solutions at different ratios.

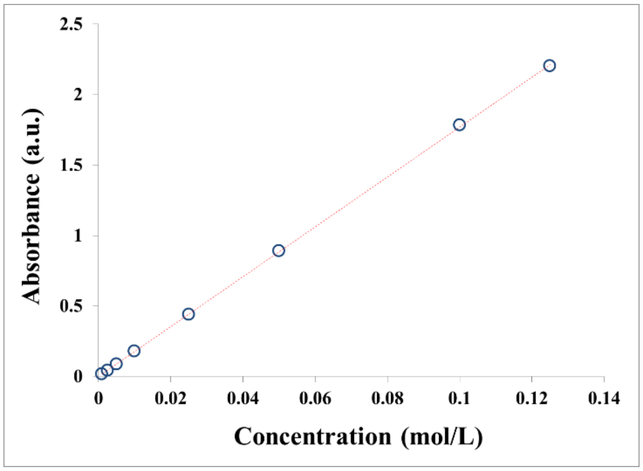

Figure S2. UV absorbance of $VOSO_4$ in 2M H_2SO_4 solutions at different concentrations.

Table S1. IEC of the investigated membranes for VRFBs.

Membranes			IEC (mmol g^{-1})	Ion	Reference
1	N212	Nafion N212	0.91	H^+	Wang, D. and C. J. Cornelius (2017). "Modeling ionomer swelling dynamics of a sulfonated polyphenylene, pentablock copolymers, and nafion." Journal of Polymer Science Part B: Polymer Physics 55(5): 435-443
2	FS-930	Fumapem FS-930	1.14	H^+	Technical Data Sheet - fumapem FS-930. Available from: https://www.fuelcellstore.com/spec-sheets/fumapem-fs-930-technical-specifications.pdf
3	DF	Dongyue DF	1.57	H^+	Drioli, E. and E. F. Macedonio (2010). "Membrane research, membrane production and membrane application in China." Available from: http://citeseerx.ist.psu.edu/viewdoc/download?doi=10.1.1.724.508&rep=rep1&type=pdf
4	VAN	VANADion™ - 20	N/A	N/A	Zhou, X.L., et al., Performance of a vanadium redox flow battery with a VANADion membrane. Applied Energy, 2016. **180**: p. 353-359.
5	FAP-450	Fumasep FAP-450	2.18	OH^-	Cho, H., et al. (2019). "Performances of anion-exchange blend membranes on vanadium redox flow batteries." Membranes 9(2): 31. Available from: https://pdfs.semanticscholar.org/282e/e9e763c970d8f15240cf9ea81c68dd08bdee.pdf
6	AHA	Neosepta AHA	0.35	Cl^-	Gopi, K. H., et al. (2014). "3-Methyltrimethylammonium poly (2, 6-dimethyl-1, 4-phenylene oxide) based anion exchange membrane for alkaline polymer electrolyte fuel cells." Bulletin of Materials Science 37(4): 877-881.
7	AMV	Selemion AMV	1.6	N/A	Lee, Y. J., et al. (2019). "Reinforced anion exchange membrane based on thermal cross-linking method with outstanding cell performance for reverse electrodialysis." RSC Advances 9(47): 27500-27509.
8	CMV	Selemion CMV	2.08	N/A	Sadrzadeh, M., et al. (2007). "Separation of different ions from wastewater at various operating conditions using electrodialysis." Separation and Purification Technology 54(2): 147-156.

Table S2. Length, width and thickness changes of membranes measured at 21°C and RH 40% in 1.6M $VOSO_4$ solution (with 2M H_2SO_4) comparing the dry membranes.

Membranes	Length changes (%)	Width changes (%)	Thickness changes (%)
**N212**	2±0	3±0	3±0
**FS-930**	0.6±0.0	0±0	3±0
**DF**	0.7±0.1	0.4±0.0	5±0
**AMV**	0±0	0.5±0.0	2±0
**CMV**	0±0	0±0	1±0
**AHA**	0.1±0.0	2±0	5±0
**FAP-450**	10±0	8±0	20±0
**VAN**	0±0	0.4±0.1	2±0
